# 763. Impact of Two-Step Testing Algorithm on Hospital-onset *Clostridioides difficile* Infections and Oral Vancomycin Prescription Practices at an Academic Medical Center

**DOI:** 10.1093/ofid/ofab466.960

**Published:** 2021-12-04

**Authors:** Mary Joyce Wingler, David A Cretella, Jason Parham, Bhagyashri Navalkele

**Affiliations:** University of Mississippi Medical Center, Jackson, MS

## Abstract

**Background:**

*Clostridioides difficile* infection (CDI) is one of the leading causes of hospital –onset (HO) infections. Clinically distinguishing true CDI versus colonization with *C.difficile* is challenging. We implemented a two-step testing algorithm to discriminate true CDI from colonization then evaluated the effect on rate of HO CDI and oral vancomycin.

**Methods:**

In May 2020, a two-step testing algorithm was implemented utilizing *C. difficile* PCR and enzyme immunoassay (EIA) glutamate dehydrogenase (Figure 1). Rates of HO CDI and use of oral vancomycin was compared in the three quarters preceding and after this intervention (July 2019-March 2020 and July 2020-March 2021, respectively). HO CDI was defined based on National Healthcare Safety Network (NHSN) Laboratory Identified (LabID) event as last positive *C.difficile* test result performed on a specimen collected >3 calendar days after admission to the facility. HO CDI rates were assessed based on Standardized Infection Ratio (SIR) data and antimicrobial use was reported in days of therapy (DoT) per 1000 patient days.

Figure 1. Two-Step Testing Algorithm for Diagnosing Clostridioides difficile infection

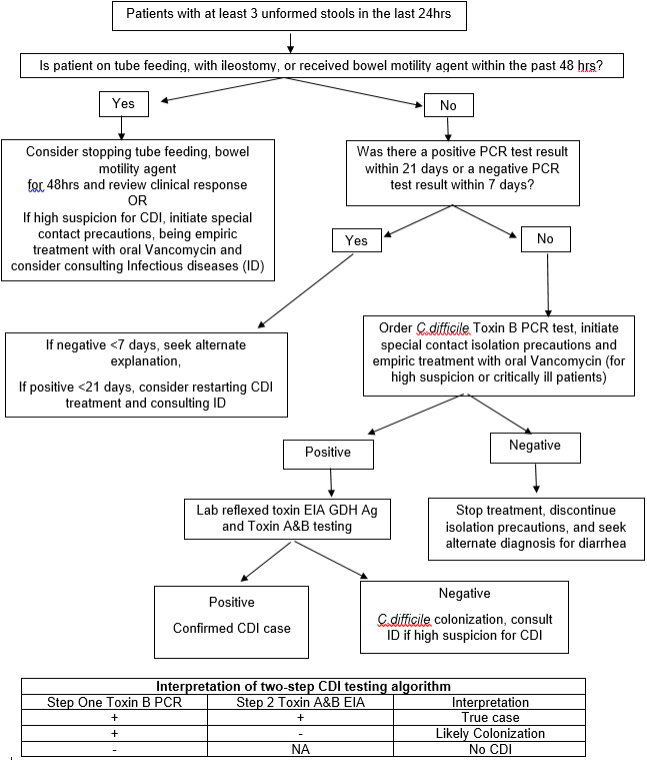

**Results:**

During the pre-intervention period 30 HO CDI cases were reported compared to 9 cases in the post-intervention period (p=0.02) (Figure 2). There was a non-statistically significant reduction in CDI SIR in post-intervention period (0.133 vs. 0.305, p=0.11). Oral vancomycin use was similar in the pre- and post-intervention periods (3.89 vs. 3.84, p=0.96). Fidaxomicin use was rare (< 0.2 DoT/1000 pt days). Of 26 HO *C.difficile* colonized patients in post-intervention period, 14 (54%) patients received oral vancomycin treatment. Infectious diseases was consulted on 7/14 and recommended discontinuation of treatment in 3 while treatment was continued for other patients based on clinical status and immunocompromising conditions.

Figure 2. Comparison of pre- and post-intervention trend in Hospital-onset CDI rate

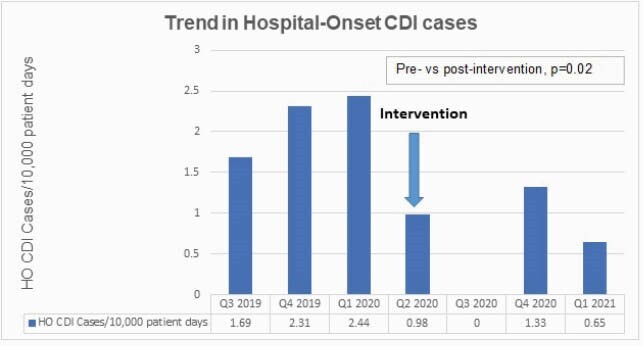

**Conclusion:**

We successfully reduced our HO CDI infections and SIR below national average after implementation of two-step testing algorithm for CDI. There was no impact on the rate of oral vancomycin use. We observed at 54% rate of treatment for patients categorized as likely colonization. Provider education and stewardship interventions are necessary to reduce inappropriate use of oral vancomycin in colonized patients.

**Disclosures:**

**All Authors**: No reported disclosures

